# Enhanced recombinant protein capture, purity and yield from crude bacterial cell extracts by *N*-Lauroylsarcosine-assisted affinity chromatography

**DOI:** 10.1186/s12934-023-02081-7

**Published:** 2023-04-25

**Authors:** Jose Vicente Carratalá, Jan Atienza-Garriga, Hèctor López-Laguna, Esther Vázquez, Antonio Villaverde, Julieta M. Sánchez, Neus Ferrer-Miralles

**Affiliations:** 1 Institut de Biotecnologia i de Biomedicina, Universitat Autònoma de Barcelona, 08193 Cerdanyola del Vallès, Barcelona Spain; 2 CIBER de Bioingeniería, Biomateriales y Nanomedicina (CIBER-BBN), Instituto de Salud Carlos III, 08193 Cerdanyola del Vallès, Barcelona Spain; 3 Departament de Genètica i de Microbiologia, Universitat Autònoma de Barcelona, 08193 Cerdanyola del Vallès, Barcelona Spain; 4 Instituto de Investigaciones Biológicas y Tecnológicas (IIBYT) (CONICET-Universidad Nacional de Córdoba), ICTA, FCEFyN, UNC., Av. Velez Sarsfield 1611, X 5016GCA Córdoba, Argentina

**Keywords:** Recombinant proteins, Difficult-to-purify proteins, *E. coli*, H6 tag, Detergent, Repurposing

## Abstract

**Background:**

Recombinant proteins cover a wide range of biomedical, biotechnological, and industrial needs. Although there are diverse available protocols for their purification from cell extracts or from culture media, many proteins of interest such as those containing cationic domains are difficult to purify, a fact that results in low yields of the final functional product. Unfortunately, this issue prevents the further development and industrial or clinical application of these otherwise interesting products.

**Results:**

Aiming at improving the purification of such difficult proteins, a novel procedure has been developed based on supplementing crude cell extracts with non-denaturing concentrations of the anionic detergent *N*-Lauroylsarcosine. The incorporation of this simple step in the downstream pipeline results in a substantial improvement of the protein capture by affinity chromatography, an increase of protein purity and an enhancement of the overall process yield, being the detergent not detectable in the final product.

**Conclusion:**

By taking this approach, which represents a smart repurposing of *N*-Lauroylsarcosine applied to protein downstream, the biological activity of the protein is not affected. Being technologically simple, the *N*-Lauroylsarcosine-assisted protein purification might represent a critical improvement in recombinant protein production with wide applicability, thus smothering the incorporation of promising proteins into the protein market.

## Background

The large-scale production of recombinant proteins has enabled their exploitation in a wide range of sectors such as biomedicine and biotechnology, for diagnostics, therapy and vaccination [1–5], as well as molecular tools in genetic engineering or catalysts in the biotech industry [3, 6]. The production of recombinant proteins, especially at large scale, suffers from important bottlenecks that minimize the yield of functional, usable products [7]. Among them, protein aggregation commonly occurs irrespective of the type of cell factory used for biofabrication [8, 9]. In bacteria, in which the recombinant proteins are in general not secreted to the media, aggregation of the recombinant protein results in large cytoplasmic structures called inclusion bodies (IBs) [10]. Soluble aggregates, probably intermediates in IB formation, also abound [11–13], since aggregation of recombinant proteins is a complex event that involves a wide spectrum of conformational conformers [14–17], ranging from properly folded versions to misfolded, amyloidal forms [16]. Globally, the initial step in recombinant protein purification is the separation, after cell lysis, of the soluble cell fraction from the insoluble cell fraction. In this scenario, the populations of recombinant protein that form IBs are retained in the insoluble cell fraction and therefore discarded from the protein purification process, that only involves soluble forms. For aggregation-prone proteins, this fact represents an immediate loss of an important portion of the product that is straightforward excluded from the purification pipeline.

However, proteins can also be recovered from IBs once these have been separated from the cell debris. In this regard, several procedures have been described for the recovery of IB protein, based on either denaturing or non-denaturing conditions. In the first, more conventional approach, the IB protein is completely denatured, solubilized, and subjected to a refolding process aiming at recovering the product in the native conformation and with full functionality [18, 19]. This methodology shows variable and highly product-dependent success rates. For non-denaturing protein extraction, which is still emerging, standard protocols have been established that allow the immediate release of IB proteins with full biological activity. This is achieved by using mild detergents like *N*-Lauroylsarcosine (N-L, also known as sarkosyl) as solubilizing agents [20]. The success of this approach is based on the important amounts of recombinant proteins with native or native-like conformations contained in the IBs [17].

Regardless of the generic optimization of protein recovery from large IB aggregates, many industrially or clinically interesting proteins fall into the category of difficult-to-purify proteins, including those that have solvent-exposed hydrophobic or cationic domains [21, 22]. Although not deeply analyzed, failure of these proteins to be purified with sufficient efficiency (e.g., by affinity chromatography) may be due to their tendency to aggregate, even as soluble versions. Consequently, there is a steric sheltering of the purification tags or the undesired acquisition of a sticky character that makes the polypeptide to interspecifically interact with other proteins. Despite the success in the application of N-L for IB protein recovery [20], this detergent had not been tested as a tool for improving the purification of soluble, difficult-to-purify protein species. In this study and by using several model proteins, we have tested the capability of N-L to favor the chromatographic purification of complex recombinant proteins from *Escherichia coli* cell extracts, keeping their biological activity and avoiding traces of the detergent in the final product. According to the presented data, we propose the repurposing of N-L from an agent for the solubilization of protein aggregates to an additive for the purification of soluble protein species and the incorporation of this detergent as a highly valuable tool for improved purification protocols to be applied to the soluble cell fraction.

## Results

**Fig. 1:**
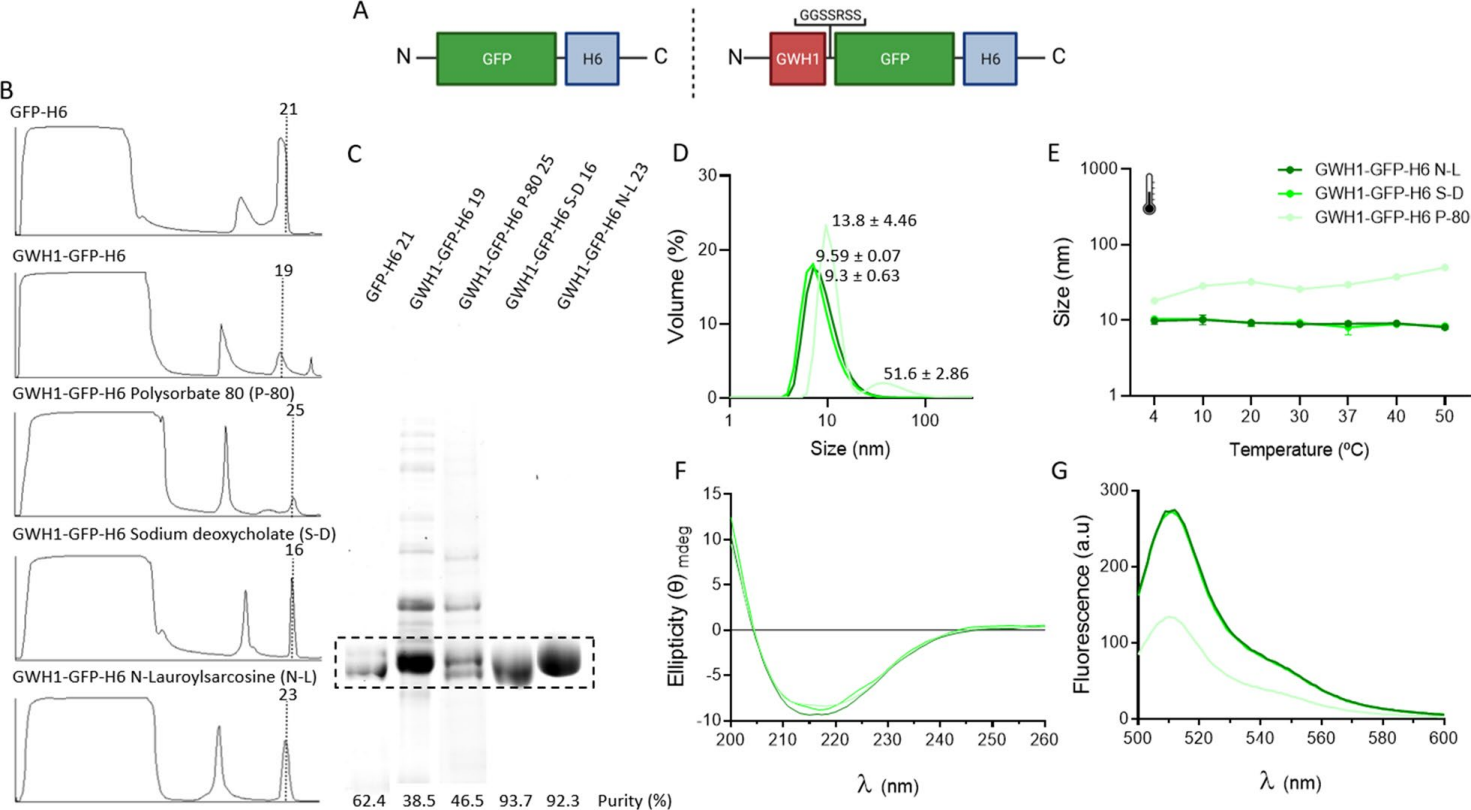
Purification of GFP-H6 and GWH1-GFP-H6. **A** Modular architecture of GFP-H6 and GWH1-GFP-H6. Box sizes are only approximate. **B** Chromatographic profile for affinity chromatography of GFP-H6 and GWH1-GFP-H6 constructs present in the *E. coli* soluble cell fraction in the absence or presence of different detergents. Vertical dashed lines and numbers indicate the fraction selected for further analysis. **C** SDS-PAGE coupled to TGX stain-free gel Technology (Bio-Rad) of the protein fractions indicated through vertical lines in the panel **B**. **D** DLS size determination of GWH1-GFP-H6 purified in the presence of alternative detergents. Numbers indicate peak sizes. **E** Temperature-dependent DLS size data of GWH1-GFP-H6 purified in the presence of alternative detergents. **F** Circular Dichroism (CD) spectra of GWH1-GFP-H6 protein samples purified in the presence of alternative detergents. **G** GFP fluorescence spectrum of GWH1-GFP-H6 purified in the presence of alternative detergents

GWH1-GFP-H6 is a modular protein (Fig. 1A, top) that contains GWH1, a cationic antimicrobial peptide (AMP) of clinical interest for the treatment of infectious diseases and in oncology [23]. The presence of the hexahistidine (H6) at the carboxy terminus of the construct allows the one-step purification of the protein by Ni^2+^-based affinity chromatography and, in addition, its self-assembling as regular homo-oligomers of around 10 nm. The oligomeric disposition of AMPs in multivalent nanoparticles is highly desirable, as such format increases the local concentration and effectiveness of the drug [24, 25]. The presence of GWH1 at the N-terminus of this construct is a clear obstacle in the downstream of the protein, as the purity of the resulting product is much lower than that of its counterpart GFP-H6 (Figure B, C). However, when different detergents are added to the cell extracts, after mechanical cell lysis and before chromatography, the purity of the products is enhanced from around 38% to more than 90% (Fig. 1C). While polysorbate 80 (P-80) had only a moderate effect (46% purity), sodium deoxycholate (S-D) and N-L were highly effective reagents. In addition, P-80 also rendered a product that appeared to be less proteolytically stable than alternative versions, since a double protein band was observed in the gels (Fig. 1C). When checking the oligomeric architecture of the proteins, S-D and N-L-managed products showed a nanometric size compatible with previous observations, in a monodisperse peak (Fig. 1D). On the contrary, when P-80 was involved, a tendency to aggregate was suspected due to the high polydispersion index showed by the protein material. Such tendency to aggregate was confirmed by increasing the temperature of the protein sample, followed by its DLS analysis (Fig. 1E). Then, a temperature-dependent increase in the size of the materials was only observed when P-80 had been involved in their recovery. The higher stability of GWH1-GFP-H6 obtained with S-D and N-L was confirmed from the structural point of view. The β structure of the protein revealed by circular dichroism was highly conserved in the three detergent strategies (Fig. 1F). However, the sample treated with P-80 exhibited a lower intensity value in the whole GFP emission fluorescence spectrum when compared to the samples treated with S-D and N-L (Fig. 1G). This result demonstrated a particular tertiary structure of GWH1-GFP-H6 obtained with P-80 that was in agreement with the high size of protein oligomers and with the observed instability (Fig. 1D, E).

**Fig. 2:**
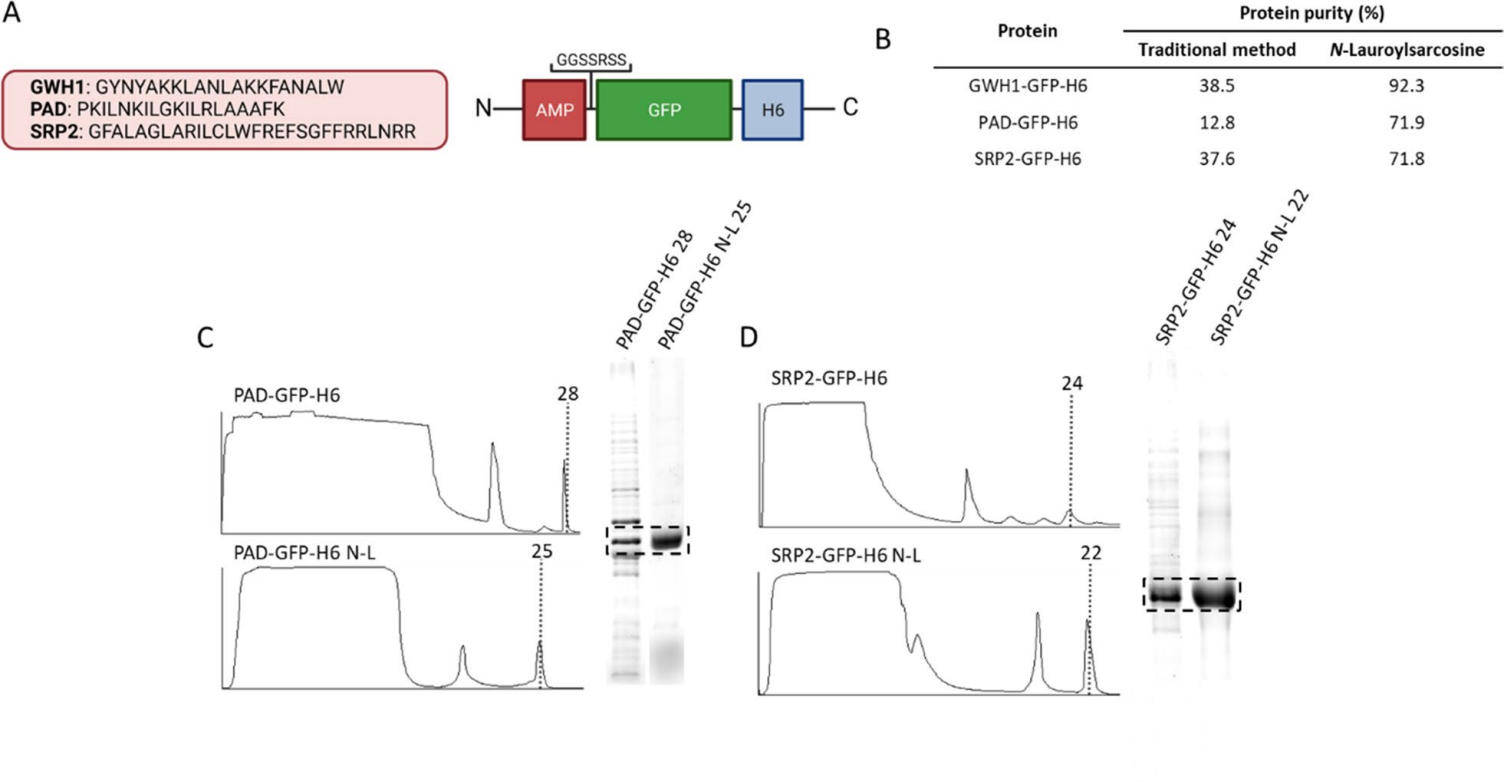
Comparative purification of cationic modular proteins based on GFP. **A** General architecture of the fusion proteins used here. Box sizes were only indicative. **B** Purity data summary. **C** and **D** Chromatographic profiles for affinity chromatography of PAD-GFP-H6 (C) and SPR2-GFP-H6 (D) from the *E. coli* soluble cell fraction in absence or presence of N-L in the cell extracts. Vertical dashed lines and numbers indicate the fraction selected for further analysis. The plots are sided by SDS-PAGE coupled to TGX stain free gel Technology (Bio-Rad) of protein fractions indicated in vertical lines

**Fig. 3:**
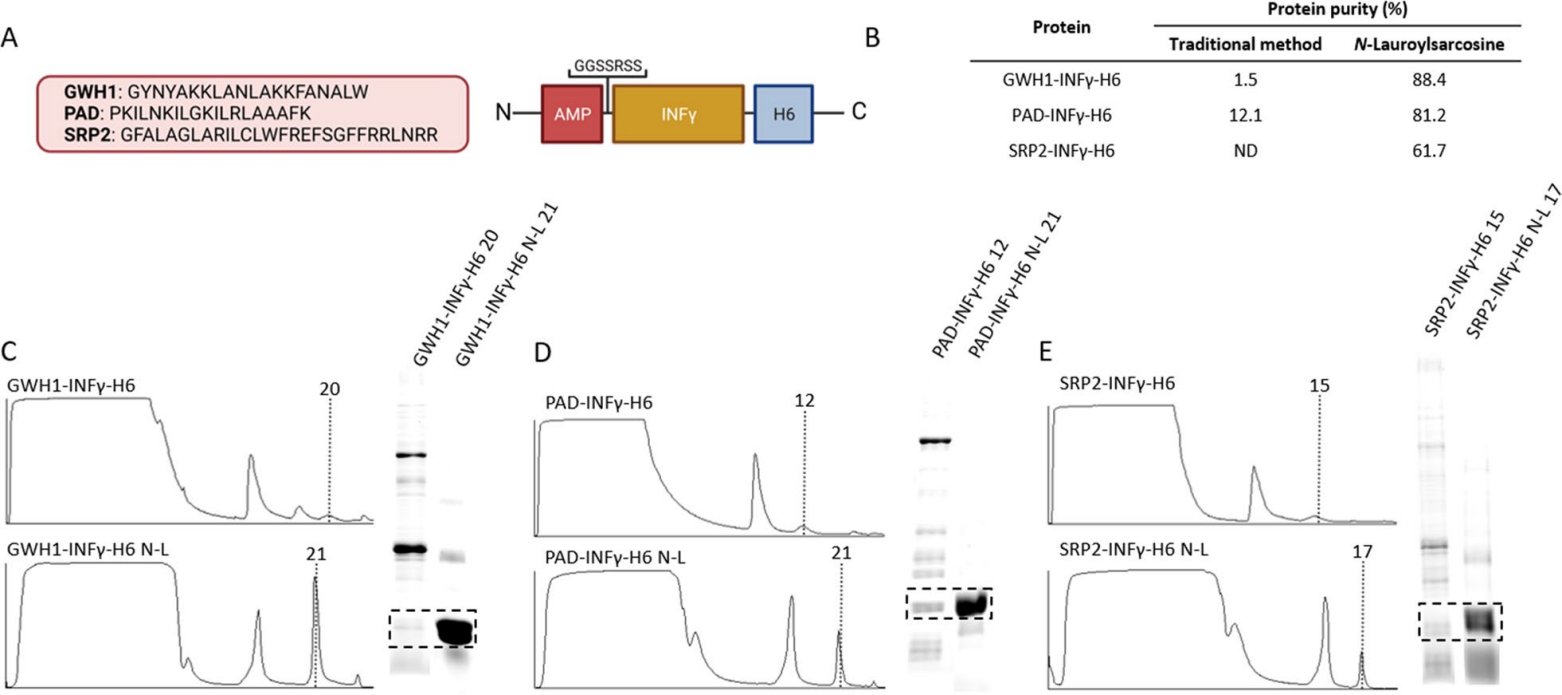
Comparative purification of cationic modular proteins based on INFγ. **A** General architecture of the fusion proteins used here. Box sizes were only indicative. **B** Purity data summary. **C**–**E** Chromatographic profiles for affinity chromatography of GWH1-INFγ-H6 (C), PAD-INFγ-H6 (D) and SPR2-INFγ-H6 from the *E. coli* soluble cell fraction in absence or presence of N-L in the cell extracts. Vertical dashed lines and numbers indicate the particular protein fraction selected for further analysis. The plots are sided by SDS-PAGE coupled to TGX stain free gel Technology (Bio-Rad) of protein fractions indicated by vertical lines

To test if the observed beneficial effect observed on protein purification might be protein-specific, two additional modular proteins based on GFP (Fig. 2A) were produced in *E. coli* and purified as described above, using N-L as an additive in the cell extracts. These proteins contained different AMPs at the N-terminus. In general, AMPs show a cationic character expected to impair purification. Therefore, being these constructs models of difficult-to-purify proteins, they were well suited for the proposed analysis. As observed (Fig. 2B), the purity of all these proteins dramatically improved when using the detergent-assisted chromatographic method for at least two-fold. Data are summarized in Fig. 2C. Again, to further discard specific links between the improved process and a particular protein domain, all these modular proteins were produced as new modular versions by substituting the scaffold GFP by a central scaffold based on the murine interferon gamma (INFγ) (Fig. 3A). The purification process of all these proteins was largely improved (Fig. 3B), and the final purity was dramatically enhanced, reaching almost 60-fold in the case of GWH1-INFγ-H6 (Fig. 3C).

**Fig. 4:**
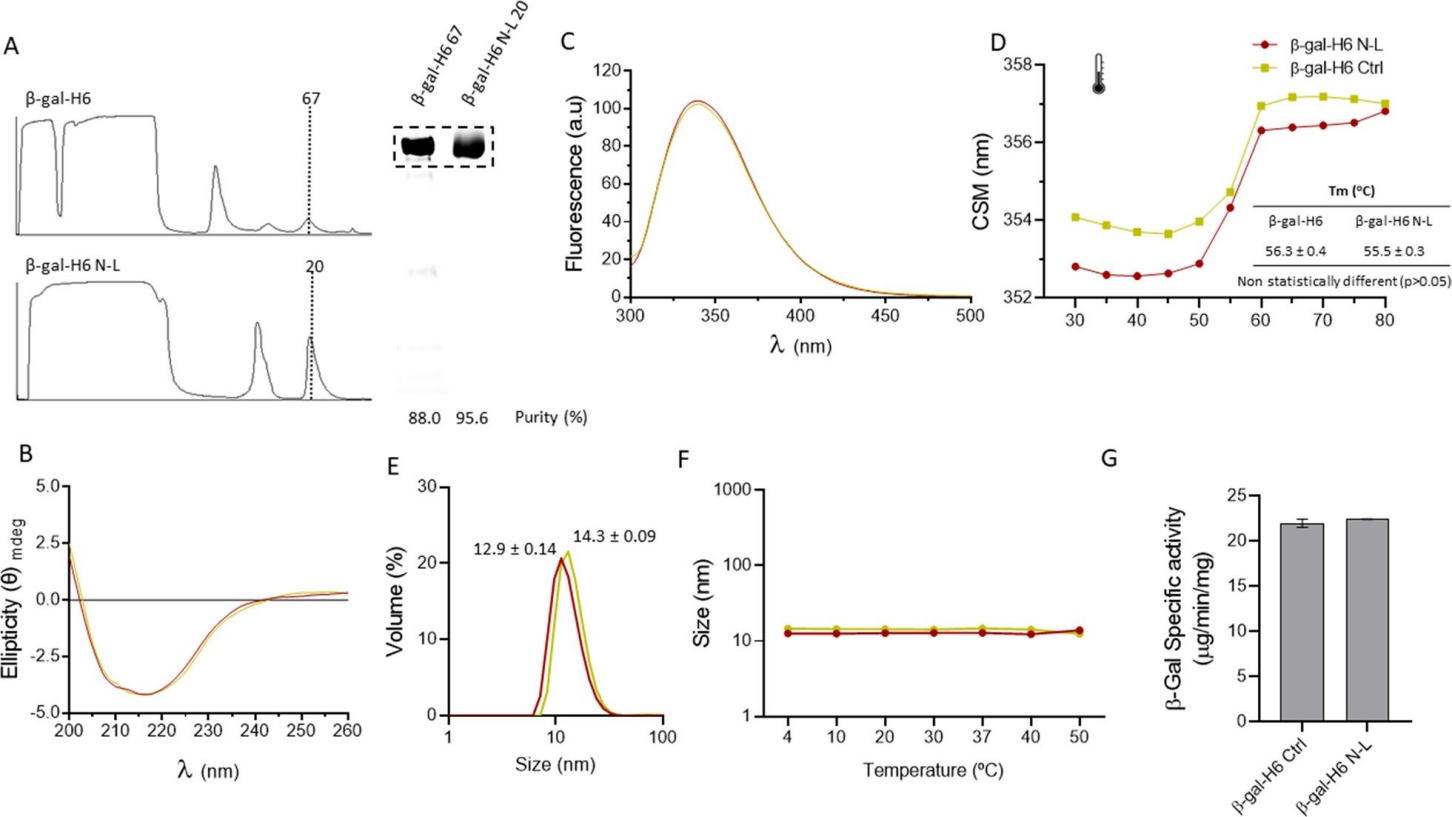
Purification of a recombinant *E. coli* β-galactosidase. **A** Comparative chromatographic profile of mAU signal at 280 nm for affinity chromatography of purification of β-gal-H6 with and without N-L. The corresponding SDS-PAGE coupled to TGX stain free gel Technology (Bio-Rad) of selected elution fractions is also shown. **B** Circular Dichroism (CD) spectra of β-gal-H6 protein samples purified with the N-L method **C** Tryptophan fluorescence spectrum of β-gal-H6 protein samples purified with N-L. **D** Center of Spectral Mass (CSM) of tryptophan fluorescence spectrum of β-gal-H6 samples purified with N-L, versus temperature. Inset: Tm values calculated from a sigmoidal model of CSM vs temperature. **E** DLS measurements of particle size distribution (by volume) of β-gal-H6 samples. **F** Thermal profile of β-gal-H6 protein size. **G** Comparative enzymatic activity of β-gal-H6 samples. Differences were not statistically different (p > 0.05)

Of course, the presence of a detergent in the cell extracts, even at low concentrations, might be a risk in a production process regarding the potential loss of conformation and functionalities of the target protein, which despite an improved yield could show a decreased biological activity. To check and to eventually discard this possibility, a conventional, his-tagged *E. coli* β-galactosidase was produced in recombinant form, and its enzymatic activity used as reporter to evaluate a potential impairment of the conformational quality. Being a tetrameric enzyme with four active sites at the protein–protein interfaces, this enzyme is sensitive to denaturing agents and any enzymatic loss should be indicative of a deleterious effect of the method. As in the case of previous tested proteins, N-L enhanced the protein purity after affinity chromatography (Fig. 4A). When testing structural parameters we noted that both protein samples, resulting from conventional purification and from detergent-assisted purification, showed indistinguishable properties. An overlap of the circular dichroism (Fig. 4B) and tryptophan fluorescence (Fig. 4C) spectra, and the DLS profiles (Fig. 4E) of both detergent-treated and untreated β-galactosidase samples confirmed that the secondary (Fig. 4B) and tertiary structures (Fig. 4C and E) of the enzyme were preserved under the tested procedure. Moreover, the thermal stability of the protein until 50 °C was unaffected (Fig. 4D and F) and there was not statistical difference between the unfolding temperature of both protein samples determined from the CMS vs temperature curve (Fig. 4D). Importantly, and according to the absence of detectable conformational modifications, the specific activity of the enzyme remained unchanged when comparing both purification protocols (Fig. 4G).

**Fig. 5:**
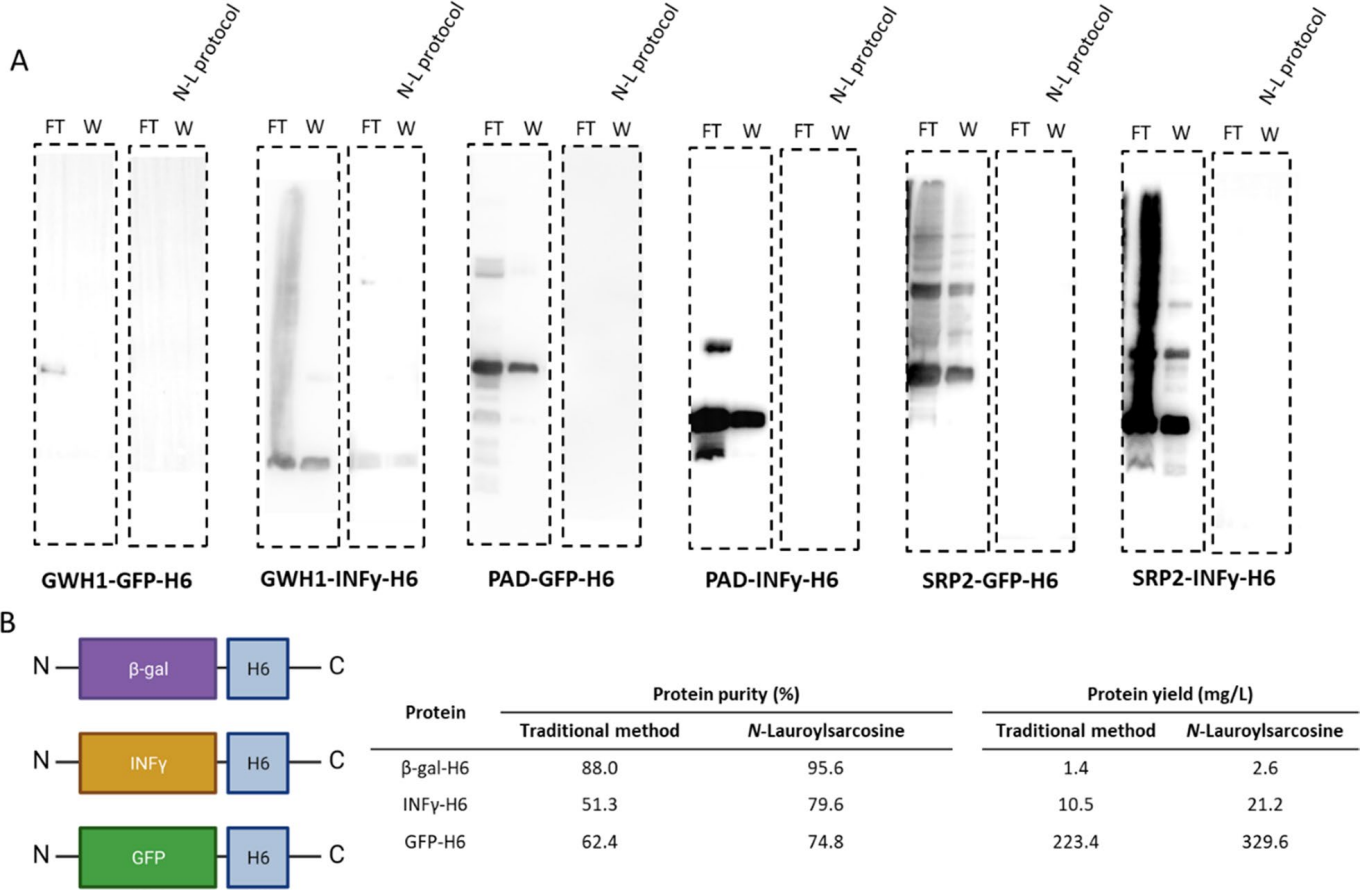
Binding of H6-tagged recombinant proteins to Ni^2^^+^ columns. **A** Western Blot (WB) of protein samples purified by the traditional protocol or assisted by N-L. For each protein, the binding efficiency of the proteins to the Ni-column is shown through the flow through (FT) and the wash (W) sample analyses. **B** General architecture of the fusion proteins used here to determine final yield. Box sizes were only indicative.** C** Summary of yield and purity data

The improved purity of all the tested proteins (Figs. 1, 2,3, 4) found upon detergent-assisted chromatographic purification might be due, as presumed, to the hindrance of heterogeneous protein interactions but also to a higher solvent-exposure of H6, expected to result in a better binding of his-tagged polypeptides to Ni^2+^ in the columns. To test this hypothesis, the amount of recombinant protein in the flow-through and wash eluates was measured for the six complex constructs produced in the study, using either the conventional purification protocol or the detergent-assisted purification method. As observed (Fig. 5A), all the tested proteins were retained by the columns with higher efficacies when the detergent was present in the extracts than when it was absent. Such high affinity can account, by itself, the enhanced purity levels observed in the final product. Of course, since high retention in the affinity columns should not only result in higher purity but in higher protein yields, this parameter was tested in the tagged proteins for which yield could be precisely estimated when using the conventional purification approach (that means, when the protocol rendered sufficient protein yields for their quantification) (Fig. 5B). As expected, for the three tagged proteins yield, in addition to purity, was systematically improved, (Fig. 5C).

**Fig. 6:**
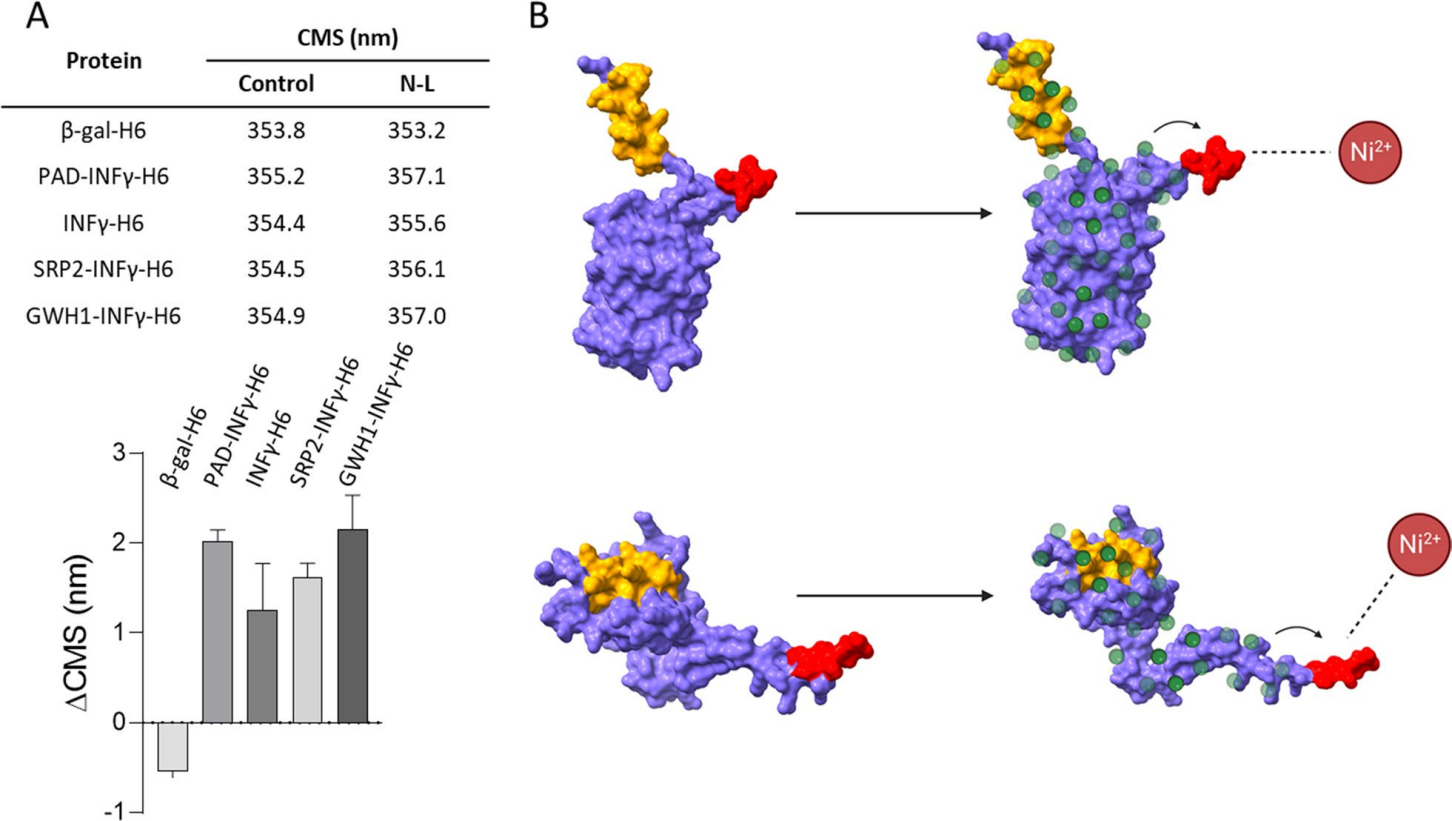
**A** Center of spectral mass (CMS) of the Trp emission (Appendix B) in the absence (control) or in the presence of N-L. These data were applied to calculate the ΔCMS = CMS_+N-L_ – CMS_control_ and all the ΔCMS values were statistically different from zero (p < 0.05). **B** Speculative model of the mechanism of action of N-L, whose presence could place the his-tag in a more convenient way for its binding to the Ni^2+^ in the column. GWH1-GFP-H6 is shown at the top and GWH1-INFγ-H6 at the bottom. Colour codes are as it follows: yellow (antimicrobial peptide), red (his-tag) purple (scaffold protein) and green (N-L molecules)

The hypothesis that the presence of detergent leads to an increased solvent exposure of H6 in recombinant proteins would be further supported if conformational changes could be detected in the protein upon incubation with N-L at the working concentrations. This possibility was tested with the set of INFγ–based proteins and the recombinant β–galactosidase. In all cases, the sets of data demonstrated that in the presence of the detergent, a negligible or a slightly change in the CMS values calculated from the Trp emission of the proteins occurred (Fig. 6A). As shown, the CMS values moved to different or higher values in most of the samples (ΔCMS > 0) in which N-L was present. This fact reflects moderate rearrangements of the protein structure mediated by N-L, that might be compatible with a better solvent-exposure of H6 in this situation than when the protein was stored in absence of the surfactant, as modelled in Fig. 6B. Importantly, N-L was not detected in the final protein samples upon the dialysis steps (Appendix A), what was of course relevant regarding the potential industry-oriented uses of the proposed protocol.

## Discussion

The final protein recovery yield in downstream recombinant protein production is a critical parameter for defining the clinical or industrial applicability of promising proteins. Importantly, many of those valuable products are discarded from industrial production and marketing because their low yield or unaffordable efforts required for purification. Apart from the seminal protein fractioning into soluble and insoluble versions, it is progressively accepted from multiple independent observations that a recombinant protein produced in bacteria occurs in a continuum of conformations, and that soluble aggregates are common in the cytoplasm or recombinant bacteria [14–16, 26]. Taking this idea as a conceptual basis, the conformational spectrum of the soluble protein versions might have a differential impact on the purification efficacies of such conformers, and at least partially, the presence of these forms might also account for the occurrence of difficult-to-purify proteins even if mostly present in the soluble cell fraction.

In this context, N-L is an anionic surfactant with multiple applications in biotechnology and biomedicine, including transdermal drug delivery [27–29], fabrication of biomaterials and nanomaterials [30–32], structural protein analysis [33, 34], separation of co-purified tag-less proteins from protein complexes [35]and mild protein solubilization/extraction [36–38], among many others. In fact, it was the first non-denaturing agent used in the solubilization of recombinant proteins from IBs [39]. This early application in the handling of IBs fully supported the concept, developed much later [40], that functional proteins could be recovered from insoluble protein aggregates by mild detergents [20], in contrast to conventional and less technologically friendly denaturing/refolding-based protocols [18].

In this line, N-L is among the most currently used agents to favor non-denaturing protein extraction from IBs, but its applicability as a generic assistant in the purification of soluble proteins had not been explored. Based on the hypothesis that at least a fraction of difficult-to-purify protein populations might have conformational defects, the use of a mild detergent might improve their downstream. This would be especially relevant regarding tag-based affinity chromatography, in which the affinity tag in the recombinant protein should be properly exposed to the solvent for a proper binding to the immobilized ligand [41–43]. We have proved here a dramatic improvement in the column retention, final purity and recovery yield of several modular proteins (Figs. 1, 2, 3, 4, 5), in which the cationic character of a fused N-terminus domain clearly impairs its downstream (compare GFP-H6 and GWH1-GFP-H6 in Fig. 1B and C). Although a compensation of charges between the cationic domain and the anionic detergent and a consequent improvement of solubility cannot be discarded, the positive effect that the surfactant shows on the purification of a conventional β-galactosidase (Fig. 4) indicates that such beneficial impact has a transversal character irrespective of the protein surface charge.

Therefore, a model has been proposed to account for the positive impact that N-L shows over H6-tagged protein absorption in Ni^2+^-based chromatography in which the detergent slightly and reversibly unfolds the protein (Fig. 6A), making H6 more solvent-exposed (Fig. 6B). This is well supported by the moderate changes in the model protein conformation reported here (Fig. 6A) as induced by N-L. Interestingly, such modifications do not affect the domain organization and probably involve local protein zones. It must be noted that the enzymatic activity of β-gal-H6, purified in absence or in presence of N-L, is indistinguishable (Fig. 4), and that the active sites of this enzyme are located in the monomer–monomer interfaces [44], making this enzyme sensitive to global conformational impacts. Also, in the line of potential obstacles for the use of N-L as a regular purification tool, the presence of detergent traces in the final product might pose severe health concerns. Indeed the presence of N-L and related surfactants in intravenous, oral, transdermal and colorectal drug formulations as protein stabilizer inhibits critical enzymes and raises several toxicological issues [28, 45–47]. Regarding the handling of N-L-treated samples, the working concentration used here (0.2%) is far below the limit of 1–30%, which corresponds to Eye Irritation 2, H319, and below the limit of > 30%, which corresponds to Skin Irritation 2, H315. Furthermore, N-L is not classified as carcinogenic, mutagenic, or toxic for reproduction, and is there are no evidence of chronic toxicity, according to the 2nd ATP of Regulation (EC) No 1272/2008 (CLP) and Directive 67/548/EEC (see https://echa.europa.eu/web/guest). Finally, N-L is not currently restricted by REACH. Regarding the final product, fine analytical methods have not detected detergent in the final samples obtained by the proposed procedure, with an analytical detection limit of 1 ppm and a global detection limit of 10 ppm for a ten-fold diluted original sample. This is achieved by conventional dialysis procedures that might be further refined if convenient. Therefore, altogether, the data presented here suggest the repurposing of N-L from a solubilizator of IB proteins to its regular use in the purification of difficult-to-purify soluble proteins by affinity methods, as a valuable supporting agent able to improve the final yield and purity of the protein up to more than 50-fold.

## Conclusions

*N*-Lauroylsarcosine is a detergent commonly used for the mild solubilization of recombinant proteins from bacterial inclusion bodies. Here we have demonstrated that the repurposing of this surfactant as a generic assistant in the affinity purification of His-tagged soluble proteins, upon its addition to the crude cell extracts, dramatically improves the binding of H6-tagged proteins to Ni^2+^. Consequently, both the yield and purity of the eluted material increases in more than one order of magnitude, resulting in improved processes and in more industrially appealing products. This approach is effective for proteins with or without cationic domains and it does not alter their biological activities. Also, upon simple dialysis, the surfactant is removed in the final sample below the detection limits of very fine analytical methods and far from any toxicologically relevant range. The structural analyses of *N*-Lauroylsarcosine-exposed soluble protein indicate light conformational adjustments that might be compatible with a subtle conformational relaxation and a higher solvent-exposure of the purification tags.

## Methods

### Strains and genes

Three different *Escherichia coli* strains were employed as hosts for the recombinant production of the different proteins constructs, namely BL21 (DE3), BL21λ Codon plus and Origami B (DE3). The modular proteins used here as models (Fig. 1A) followed the same architectonic principle. From N-terminus to C-terminus; an antimicrobial peptide (AMP), a peptidic linker (GGSSRSS), a scaffold protein (either GFP or INFγ) and a H6-Tag. In GFP-H6, no AMP was present at the N-terminus. All DNA sequences were synthetized by GeneArt (Waltham, MA, USA) and codon-optimized for *Escherichia coli*. The DNA segments encoding INFγ-H6 (mouse interferon gamma), GFP-H6, GWH1-GFP-H6, GWH1-INFγ-H6, PaDBS1R1-GFP-H6 (abbrev. PAD-GFP-H6) [48], PAD-INFγ-H6, SRP2-GFP-H6 [49] and SRP2-INFγ-H6 were cloned into pET22b (Amp^R^). On the other hand, a β-galactosidase-H6 (β-gal-H6) encoding gene was cloned into pET26b (Kan^R^). The different vectors containing the synthetic genes were transformed by heat shock (42 °C for 45 s) in chemically competent *Escherichia coli* BL21 (DE3) cells for the production INFγ-H6, GWH1-GFP-H6, GWH1- INFγ-H6, PAD-GFP-H6, PAD-INFγ-H6, SRP2-GFP-H6 and SRP2-INFγ-H6, *Escherichia coli* BL21λ Codon plus for the production of β-gal-H6 or *Escherichia coli* Origami B (DE3) for the production of GFP-H6. Protein sequences are indicated in the Appendix C.

### Gene expression and recombinant protein production

All *E. coli* cultures (1–2 L) were grown at 37 °C and 250 rpm in LB broth with ampicillin at 100 μg/ml or kanamycin at 34 μg/ml (only for β-gal-H6 production). *E. coli* Origami B (DE3) cultures were grown in LB with ampicillin at 100 μg/ml, kanamycin at 34 μg/ml and tetracycline at 12.5 μg/ml. Once an optical density (OD_600_) of around 0.5 was reached, isopropyl β-d-1-thiogalactopyranoside (IPTG) was added at 0.1 mmol/L and the expression temperature set at 20 °C for and overnight culture. Cells were harvested by centrifugation (5000*g* for 15 min at 4 °C) and stored at − 80 °C.

### Protein purification protocols

For protein purification, the cell pellet was resuspended in wash buffer (Buffer A = 40 mM Tris HCl (pH 8) / 500 mM NaCl) with the protease inhibitor complete EDTA-free (Roche). Bacterial cell lysis was performed by high-pressure homogenization using the Avestin Emulsiflex C5 (ATA scientific). After the disruption, the cellular lysate was divided into aliquots of approximately 25 ml. To each one of these aliquots a specific volume of a solution containing 40 mM Tris HCl (pH 8) and 500 mM NaCl, 2% N-L was added so that the final concentration of N-L was 0.2%. The sample containing the detergent was incubated at room temperature with little agitation during 15–18 h. The next day, the soluble and insoluble fractions were separated by centrifugation (45 min, 15,000*g* at 4 °C) and the soluble fraction was filtrated firstly through a 0.45 µm filter and then through a 0.22 µm filter (Millex®-GP, Millipore Express ® PES Membrane Filter Unit). Recombinant proteins were purified in an Äkta Pure FPLC system (GE Healthcare) by immobilized metal affinity chromatography (IMAC). After selective binding to a HisTrap HP 5 ml column (GE Healthcare), proteins were eluted by a linear gradient of elution buffer (40 mM Tris HCl (pH 8), 500 mM NaCl, 500 mM Imidazol and 0.2% N-L). The eluted fractions were analyzed through Mini-PROTEAN TGX Stain-Free Gels and Western blot. The selected protein fractions were dialyzed against sodium bicarbonate salt buffer (166 mmol/L NaCO_3_H and 333 mmol/L NaCl, pH 8.0) or bicarbonate buffer (166 mmol/L NaCO_3_H, pH 8.0) and the final protein concentration was determined by NanoDrop One Microvolume UV–Vis Spectrophotometer (Thermo Scientific). The regular purification protocol does not include the presence of the detergent N-L and the overnight incubation at room temperature.

### Dynamic light scattering

The volume size distribution (expressed in nm) of all protein candidates was determined by Dynamic Lights Scattering (DLS) at 633 nm and both standard (25 °C) and increasing temperatures (from 4 to 50 °C) in a Zetasizer Advance Pro (Malvern Panalytical) using a ZEN2112 3 mm quartz cuvette. Samples were measured at least in triplicate, gaussian curves represented, and data expressed as mean ± standard error respectively.

### Fluorescence measurements

Fluorescence spectra were obtained with a Cary Eclipse spectrofluorometer (Agilent Technologies) with a quartz cell of 2 mm path length.

### Trp fluorescence

In proteins other than GFP we determined the tryptophan (Trp) fluorescence. The excitation and emission slit were set at 5 nm. The excitation wavelength (λ_ex_) was 295 nm. Fluorescence emission spectra were acquired within a range from 300 to 500 nm. To evaluate the effect of N-L on the Trp emission, 0.2% of N-L was added to each protein sample. To analyse the thermal stability of proteins each spectrum was acquired in an increasing temperature range (from 25 to 80ºC) and the temperature of unfolding (Tm) was calculated from the center of spectral mass (CSM) vs temperature curve as described elsewhere [50]. For GFP versions, the excitation slit was set at 2.5 nm and the emission slit at 5 nm. The excitation wavelength (λ_ex_) was 488 nm. The fluorescence emission spectra were acquired within a range from 500 to 600 nm.

### Circular dichroism

Data were collected in a Jasco J-715 spectropolarimeter (JASCO, Oklahoma City, OK, USA) with a thermostatic device by a Peltier system spectropolarimeter, using a 0.2 mm path length quartz cell. Each spectrum was an average of seven scans. The protein concentration was 0.15 mg/ml in each protein buffer. CD spectra were collected from 260–200 nm. Each final spectrum was obtained from three replicas. Finally, we applied a negative exponential equation for the data smoothing.

### Determination of β-galactosidase enzymatic activity

β -Galactosidase activity was measured in PBS 1X by monitoring the colorimetric signal at 420 nm produced by the degradation of an artificial substrate, o-nitrophenyl-β-D-galactopyranoside (ONPG) [51].

### Detection of N-L in protein samples by mass spectrometry

N-L in dialyzed protein samples was determined using mass spectrometry by the Chemical Analysis Service (SAQ), a technological unit at the Universitat Autònoma de Barcelona (UAB). For that, an HPLC system was used equipped with a DAD detector from Agilent Technologies and a micrOTOF-Q (time-of-flight) mass spectrometer from Bruker Daltonics. Electrospray ionization in negative polarity was used to register the surfactant. 10 µL of samples were injected using water:acetonitrile (ACN) (1:1) at 0.1 ml/min to the mass spectrometer. The parameters were adjusted for sample injection from the liquid chromatograph using Flow Injection Analysis (FIA) and detection in the mass spectrometer, without using any chromatographic column, and taking a 20 ppm N-L standard to obtain maximum instrumental sensitivity. The mass spectrometer was focused on low masses (m/z < 1000), given the molecular weight of the surfactant (m/z = 270.2), and the registrations were performed in negative polarity. To ensure that there was no carryover between protein samples, the injection was changed to positive polarity, and the protein was then injected. Data analysis indicated that there was no accumulation of protein in the instrument. Protein samples were diluted 1:10 in water to reduce the carbonate concentration and to make it compatible with the operational conditions of the instrument. Control, N-L-enriched samples were prepared using the same procedure but adding N-L in the vial to 1 ppm. This method allows then the detection of 1 ppm of surfactant per vial, that for ten-fold diluted samples makes a detection limit of 10 ppm per protein sample.

### Protein design and Three-Dimensional (3D) structure prediction

The 3D structures of the stable folded state of GWH1-GFP-H6 and GWH1-INFγ-H6 were predicted in silico using the AlphaFold2 [52] algorithm integrated in ColabFold [53] and using the default settings after introducing each primary FASTA sequence as query, respectively. Molecular graphics and analyses were performed with UCSF Chimera, developed by the Resource for Biocomputing, Visualization, and Informatics at the University of California, San Francisco, with the support from NIH P41-GM103311 [54].

### Statistical analysis

All statistical analyses were conducted in GraphPad Prism version 8.0.0 for Windows (GraphPad Software, San Diego, CA, USA) with at least two independent replicates unless otherwise indicated. T tests to assess the differences in Tm values or in the specific enzymatic activity values were applied assuming unequal variances. All quantitative values were expressed as mean ± standard error of the mean. The significance of the statistical difference was included in each experiment. A nonlinear regression analysis was developed to determine the melting temperature (Tm) of β-gal-H6 in both conditions from a sigmoidal model.

## Data Availability

The dataset supporting the conclusions of this article is available at https://doi.org/10.34810/data616

## Appendices

### Appendix A

Analysis of N-L in dialyzed protein samples.

Superimposed Extracted Ion Chromatograms (EICs) at m/z = 270.2 of a purified β-Gal-H6 sample (grey) and an equivalent sample in presence of added 1 ppm N-L (blue).

### Appendix B

Tryptophan emission spectra of non GFP proteins (0.2 mg/ml) in the presence or absence of 0.2% N-L.

### Appendix C

Amino acid sequences of the proteins used in the present study.

- eGFP-H6 MSKGEELFTGVVPILVELDGDVNGHKFSVSGEGEGDATYGKLTLKFICTTGKLPVPWPTLVTTLTYGVQCFSRYPDHMKRHDFFKSAMPEGYVQERTISFKDDGNYKTRAEVKFEGDTLVNRIELKGIDFKEDGNILGHKLEYNYNSHNVYITADKQKNGIKANFKIRHNIEDGSVQLADHYQQNTPIGDGPVLLPDNHYLSTQSALSKDPNEKRDHMVLLEFVTAAGITHGMDELYKHHHHHH
- INFɣ-H6 MAQGQFFREIENLKEYFNASSPDVAKGGPLFSEILKNWKDESDKKIIQSQIVSFYFKLFENLKDNQVIQRSMDIIKQDMFQKFLNGSSEKLEDFKKLIQIPVDDLQIQRKAINELIKVMNDLSPKSNLRKRKRSQNLFRGRRASTKHHHHHH
- GWH1-GFP-H6 MGYNYAKKLANLAKKFANALWGGSSRSSSKGEELFTGVVPILVELDGDVNGHKFSVSGEGEGDATYGKLTLKFICTTGKLPVPWPTLVTTLTYGVQCFSRYPDHMKRHDFFKSAMPEGYVQERTISFKDDGNYKTRAEVKFEGDTLVNRIELKGIDFKEDGNILGHKLEYNYNSHNVYITADKQKNGIKANFKIRHNIEDGSVQLADHYQQNTPIGDGPVLLPDNHYLSTQSALSKDPNEKRDHMVLLEFVTAAGITHGMDELYHHHHHH
- GWH1-INFɣ-H6 MGYNYAKKLANLAKKFANALWGGSSRSSHGTVIESLESLNNYFNSSGIDVEEKSLFLDIWRNWQKDGMKILQSQIISFYLRLFEVLKDNQAISNNISVIESHLITTFFSNSKAKDAFMSIAKFEVNNPQVQRQAFNELIRVVQLLPESSLRKRKRSRCHHHHHH
- PaDBS1R1-GFP-H6 MPKILNKILGKILRLAAAFKGGSSRSSSKGEELFTGVVPILVELDGDVNGHKFSVSGEGEGDATYGKLTLKFICTTGKLPVPWPTLVTTLTYGVQCFSRYPDHMKRHDFFKSAMPEGYVQERTISFKDDGNYKTRAEVKFEGDTLVNRIELKGIDFKEDGNILGHKLEYNYNSHNVYITADKQKNGIKANFKIRHNIEDGSVQLADHYQQNTPIGDGPVLLPDNHYLSTQSALSKDPNEKRDHMVLLEFVTAAGITHGMDELYHHHHHH
- PaDBS1R1-INFɣ-H6 MPKILNKILGKILRLAAAFKGGSSRSSHGTVIESLESLNNYFNSSGIDVEEKSLFLDIWRNWQKDGDMKILQSQIISFYLRLFEVLKDNQAISNNISVIESHLITTFFSNSKAKDAFMSIAKFEVNNPQVQRQAFNELIRVVHQLLPESSLRKRKRSRCHHHHHH
- SRP2-GFP-H6 MGFALAGLARILCLWFREFSGFFRRLNRRFAMRRRGGSSRSSSKGEELFTGVVPILVELDGDVNGHKFSVSGEGEGDATYGKLTLKFICTTGKLPVPWPTLVTTLTYGVQCFSRYPDHMKRHDFFKSAMPEGYVQERTISFKDDGNYKTRAEVKFEGDTLVNRIELKGIDFKEDGNILGHKLEYNYNSHNVYITADKQKNGIKANFKIRHNIEDGSVQLADHYQQNTPIGDGPVLLPDNHYLSTQSALSKDPNEKRDHMVLLEFVTAAGITHGMDELYHHHHHH
- SRP2-INFɣ-H6 MGFALAGLARILCLWFREFSGFFRRLNRRFAMRRRGGSSRSSHGTVIESLESLNNYFNSSGIDVEEKSLFLDIWRNWQKDGDMKILQSQIISFYLRLFEVLKDNQAISNNISVIESHLITTFFSNSKAKDAFMSIAKFEVNNPQVQRQAFNELIRVVHQLLPESSLRKRKRSRCHHHHHH
- β-gal-H6 GSHMLEDPVVLQRRDWENPGVTQLNRLAAHPPFASWRNSEEARTDRPSQQLRSLNGEWRFAWFPAPEAVPESWLECDLPEADTVVVPSNWQMHGYDAPIYTNVTYPITVNPPFVPTENPTGCYSLTFNVDESWLQEGQTRIIFDGVNSAFHLWCNGRWVGYGQDSRLPSEFDLSAFLRAGENRLAVMVLRWSDGSYLEDQDMWRMSGIFRDVSLLHKPTTQISDFHVATRFNDDFSRAVLEAEVQMCGELRDYLRVTVSLWQGETQVASGTAPFGGEIIDERGGYADRVTLRLNVENPKLWSAEIPNLYRAVVELHTADGTLIEAEACDVGFREVRIENGLLLLNGKPLLIRGVNRHEHHPLHGQVMDEQTMVQDILLMKQNNFNAVRCSHYPNHPLWYTLCDRYGLYVVDEANIETHGMVPMNRLTDDPRWLPAMSERVTRMVQRDRNHPSVIIWSLGNESGHGANHDALYRWIKSVDPSRPVQYEGGGADTTATDIICPMYARVDEDQPFPAVPKWSIKKWLSLPGETRPLILCEYAHAAGNSLGGFAKYWQAFRQYPRLQGGFVWDWVDQSLIKYDENGNPWSAYGGDFGDTPNDRQFCMNGLVFADRTPHPALTEAKHQQQFFQFRLSGQTIEVTSEYLFRHSDNELLHWMVALDGKPLASGEVPLDVAPQGKQLIELPELPQPESAGQLWLTVRVVQPNATAWSEAGHISAWQQWRLAENLSVTLPAASHAIPHLTTSEMDFCIELGNKRWQFNRQSGFLSQMWIGDKKQLLTPLRDQFTRAPLDNDIGVSEATRIDPNAWVERWKAAGHYQAEAALLQCTADTLADAVLITTAHAWQHQGKTLFISRKTYRIDGSGQMAITVDVEVASDTPHPARIGLNCQLAQVAERVNWLGLGPQENYPDRLTAACFDRWDLPLSDMYTPYVFPSENGLRCGTRELNYGPHQWRGDFQFNISRYSQQQLMETSHRHLLHAEEGTWLNIDGFHMGIGGDDSWSPSVSAEFQLSAGRYHYQLVWCQKLGHHHHHH
